# Discriminating Analysis of Metal Ions via Multivariate Curve Resolution–Alternating Least Squares Applied to Silver Nanoparticle Sensor

**DOI:** 10.3390/nano15010057

**Published:** 2025-01-02

**Authors:** Andrea Rossi, Massimiliano Cuccioloni, Francesco Pellegrino, Rita Giovannetti, Eugenio Alladio

**Affiliations:** 1 Department of Chemistry and NIS Centre, University of Torino, Via Giuria 7, 10125 Torino, Italy or andrea.rossi@unicam.it (A.R.); francesco.pellegrino@unito.it (F.P.); eugenio.alladio@unito.it (E.A.); 2 School of Science and Technology, Chemistry Division, University of Camerino, 62032 Camerino, Italy; rita.giovannetti@unicam.it; 3 School of Biosciences and Veterinary Medicine, University of Camerino, 62032 Camerino, Italy

**Keywords:** MCR-ALS, silver nanoparticle sensor, chemometric analysis, metal ions sensing

## Abstract

Heavy metals are life-threatening pollutions because of their great toxicity, long-term persistence in nature and their bioaccumulation in living organisms. In this work, we performed multivariate curve resolution–alternating least squares analysis of UV-Vis raw spectra received by a colorimetric sensor constructed on mercaptoundecanoic acid functionalized silver nanoparticles (AgNPs@11MUA) to detect Cd^2+^, Cu^2+^, Mn^2+^, Ni^2+^, and Zn^2+^ in water. This combined approach allowed the rapid identification and quantification of multiple heavy metals and showed adequate sensitivity and selectivity, thus representing a promising analytical and computational method for both laboratory and field applications such as environmental safety and public health monitoring.

## 1. Introduction

*Background:* In recent years, chemometric techniques have become very attractive within the scientific community since they significantly enhance the ability to analyze complex datasets and extract reliable information [1]. The methodologies use statistical and mathematical principles to effectively interpret, validate, and quantify chemical data. The application of chemometric techniques is particularly useful in the field of environmental monitoring due to the high complexity of chemical systems such as spectral overlap, noise, and the presence of multiple components in samples [2]. Many studies are focused on the development of colorimetric sensors for analyte determination [3,4,5,6]. These sensors enable the rapid, real-time, sensitive, and selective detection of heavy metals, which is crucial for monitoring health problems associated with heavy metal ions such as skin disorders, liver damage, kidney disfunction, cardiac symptoms, neurological impairment and cancer [7,8,9,10].

*Nanoparticle-based colorimetric sensors:* These types of sensors are generally made of gold or silver nanoparticles [11,12,13], and their basic mechanism is based on the interaction between the target metal ion and the functionalizing agent that covers the nanoparticle: this binding event triggers the aggregation of the nanoparticles and induces changes to their optical properties, which can be exploited for the detection and/or the quantification of the analyte [4,14]. Specifically, the interaction of nanoparticles with target analytes causes alterations in interparticle distance and plasmon coupling, inducing significant shifts in the surface plasmon resonance (SPR) peak and in the color of the solution [15]. The surface chemistry of AgNPs plays a key role in the aggregation mechanism; specific ligands are often employed to modify their surfaces, enhancing stability and selectivity towards analytes [16,17,18]. Huge efforts are dedicated to the selection of a functionalizing agent that selectively interacts with target molecules, avoiding interferences with molecules that have similar physical–chemical properties to the target molecule. These functionalizing ligands are generally bi-functional organic molecules with a head that stably binds to the nanoparticle surface and a tail that will interact with the analyte. Thiol/thiolate ligands are commonly used for this purpose since they can strongly interact with silver or gold surfaces, generating a covalent interaction and influencing the nanoparticles’ aggregation behavior and, consequently, their optical responses [19,20,21]. Although these sensors do not match the performance of instrumental analytical techniques, they offer advantages such as lower costs, ease of use, and reduced waiting times for results [22,23,24,25,26,27]. The literature reports several studies that detail the development of colorimetric sensors for the determination of metal ions, often achieving good detection limits [28]. Nevertheless, many of these sensors are specific to a single metal ion, necessitating multiple colorimetric sensors based on nanoparticles for investigations involving multiple metal ions [16].

*Chemometric analyses:* To try to address this issue, we combined a functionalized silver nanoparticle-based sensor with multivariate curve resolution–alternating least squares (MCR-ALS) analysis, a versatile chemometric technique suitable for the analysis of complex systems using colorimetric sensors [29,30], which are often affected by overlapping spectral responses generated by mixtures of analytes. In fact, MCR-ALS allows the deconvolution of overlapping signals, eventually providing quantitative and qualitative information on the chemical species present in the mixture of interest. Regarding colorimetric sensors, MCR-ALS offers several advantages such as the ability to handle spectroscopic data with overlapping absorption bands, resolve contributions from multiple analytes, and operate upon minimal training. It can also improve the accuracy, sensitivity, and selectivity of colorimetric detection methods applied to multiple analytes or complex matrices. Noteworthy in this context, MCR-ALS has been successfully applied to analyze environmental samples, food safety assays, and biomedical diagnostics, where the deconvolution of mixed signals is critical for the accurate detection and quantification of target species [31,32,33].

Upon training on the spectra obtained for individual metal ions, the proposed chemometric approach allowed us to effectively discriminate the overlapping UV-Vis signals obtained from our sensor in the presence of a panel of the most common (and most prone to bioaccumulation) metal ions [34,35], namely Ni^2+^, Zn^2+^, Co^2+^, Cd^2+^, Mn^2+^, and Cu^2+^. Globally, MCR-ALS applied to the obtained spectroscopic data provided an accurate identification of each analyte using functionalized AgNPs over a specific range of concentrations.

## 2. Materials and Methods

**Materials:** AgNO_3_, NaBH_4_, mercaptoundecanoic acid (11MUA), NiCl_2_, CoCl_2_, ZnCl_2_, CuCl_2_, MnCl_2_, and CdCl_2_ were purchased from Sigma-Aldrich (St. Louis, MO, USA). All chemicals were used as received without further purification. Aqua regia was used to clean all the glassware prior to use. Ultrapure water (18.2 µS/cm), produced by a Milli-Q^®^ Advantage A10 system from Merk, was used for the preparation of all solutions.

**The synthesis and characterization of functionalized AgNPs:** The functionalized AgNPs were synthesized using a two-step process as previously reported [36]. Briefly, silver nanoparticles (AgNPs) were formed through a reduction reaction. Then, the surface of the AgNPs was functionalized by adding a specific amount of 11MUA to obtain a partial surface functionalization [36], followed by prolonged stirring to ensure a proper interaction between the AgNPs surface and functionalizing ligand. This particular sensor was used throughout. The UV-Vis spectra of both bare AgNPs and AgNPs functionalized with 11MUA (AgNPs@11MUA) were recorded using a Cary 8454 Diode Array spectrophotometer (Agilent, Santa Clara, CA, USA), with surface plasmon absorption bands (SPABs) observed at each titration step. The colorimetric response of the AgNPs@11MUA sensor was then evaluated by stepwise additions of 10 µL of metal stock solutions (0.1 mM) into the suspension, recording the SPAB at each step to construct dose/response curves.

**Data Handling:** MCR-ALS analysis is a powerful tool used to deconvolute complex data into their pure components (i.e., absorbance spectra and concentration profiles) [37,38,39,40], and it has been extensively applied in UV-Vis spectroscopy [31,41,42,43,44]. In fact, MCR-ALS allows the handling of overlapping signals, the resolution of unknown components without prior standards, and the adaptation to non-linear or constrained modeling scenarios. These characteristics make it particularly suitable for the multivariate data analysis of complex systems, such as bio-analytical sensor development for environmental monitoring [23,45]. Furthermore, the implementation in MATLAB toolboxes [40] and Python libraries like *SpectroChemPy* has simplified its accessibility, allowing researchers from diverse disciplines to integrate it into their workflows [46].

MCR-ALS is particularly effective for measurements that follow Lambert–Beer’s law, where the overall spectra collected are a linear combination of the spectra of their pure components. The following equation can summarize the deconvolution process:

(1) $$M={CS}^{T}+E$$

In this equation, *M* represents the collected data matrix (SPABs), *C* is the matrix of pure concentration profiles, *S^T^* is the transposed matrix of pure spectra, and *E* is the residuals (error) matrix.

Consequently, each collected spectrum *m* in the *M* matrix can be expressed as follows:

(2) $$x=C_{1}S_{1}^{T}+C_{2}S_{2}^{T}+\ldots +C_{n}S_{n}^{T}$$

where *C*_1_, …, *C_n_*, represent the concentrations of the n pure components or chemical species for the evaluated mixture, while *S*_1_, …, *S_n_*, denote the spectra of these pure components. This framework can be extended to all collected spectra within the *M* matrix.

The first step in the MCR-ALS deconvolution involves the initial estimation of either pure concentration matrix *C* or pure spectra matrix *S* through several algorithms [47,48,49,50]. Once the initial estimate is obtained, the initial *C* matrix is computed through ALS by minimizing the residuals, *E*. This iterative process continues until an optimal convergence value is achieved. Additionally, various constraints can be applied in MCR-ALS calculations based on the chemical and physical properties of the samples and the data under investigation [48].

During the analysis of UV-Vis spectra by means of MCR-ALS, several constraints can be applied to enhance robustness and accuracy. The *non-negativity constraint* ensures that concentration profiles and spectra contain no negative values, maintaining physical validity. The *closure constraint* upholds mass balance, ensuring the total concentration of all components remains constant, which is crucial in stoichiometrically controlled reactions and biological mixtures. The *unimodality constraint* enforces a single peak in the concentration profile, reflecting predictable patterns such as chromatographic elution profiles. The *normalization constraint* standardizes spectra or concentration profiles, facilitating consistent comparisons across datasets. Finally, the *angle constraint* improves the resolution of closely related components by enhancing contrast, enabling better spectral and concentration discrimination [51]. Chemometric analyses were performed with R version 4.3.1 (R Core Team, 2024) and MatLab R2024b (the MathWorks Inc. 2024, Natick, MA, USA).

## 3. Results and Discussions

We previously demonstrated that an aqueous suspension of AgNPs (yellow) undergoes a color shift upon the addition of metallic ions in the form of chloride salts [18]. This color change is caused by the aggregation of two or more nanoparticles, which occurs because of interactions between the carboxylic acid groups present on the nanoparticle surfaces and the metallic ions. The nature of these interactions depends primarily on the characteristics of the metallic ion—such as its coordination number, solvation sphere, and atomic number—as well as the density of surface functionalization of the nanoparticles [36]. Most importantly, each metallic ion triggers the formation of macroaggregates (or superlattices) with peculiar shapes and sizes, which results in the appearance of distinct colors at high (pre-collapsing) metal ion concentrations, with distinctive titration curves, linearity ranges and LOD/LOQ values (see Supplementary Materials).

**Figure 1:**
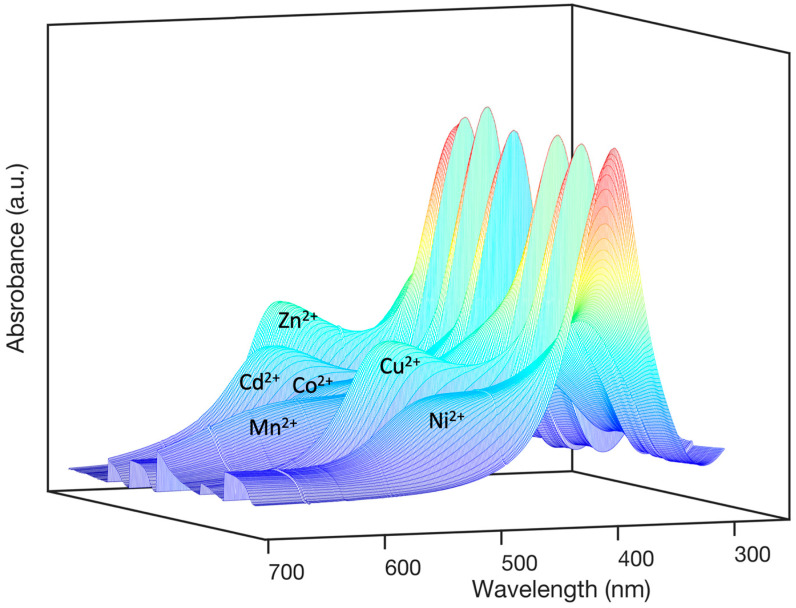
Representative 3D comparison of UV-VIS spectra obtained upon individual titration of AgNPs@11MUA with Ni^2+^ [0–14.5 μM], Cu^2+^ [0–10.5 μM], Mn^2+^ [0–20 μM], Co^2+^ [0–12 μM], Cd^2+^ [0–9.5 μM], and Zn^2+^ [0–18 μM] at 20 °C and atmospheric pressure.

Figure 1 shows a 3D comparison of UV-VIS spectra obtained upon the individual titration of AgNPs@11MUA with each metal. As expected, a reduction in the absorbance of the SPAB specific to AgNPs@11MUA was observed, along with the appearance of a second metal-type-specific SPAB. The second band can be attributed to various factors, including plasmonic coupling, changes in the interparticle distance, near-field interactions, aggregation, and alterations in the shape of the silver nanoparticles [15]. These phenomena occur when the local electric fields of two or more silver nanoparticles overlap and interact as their interparticle distance decreases [52].

**Figure 2:**
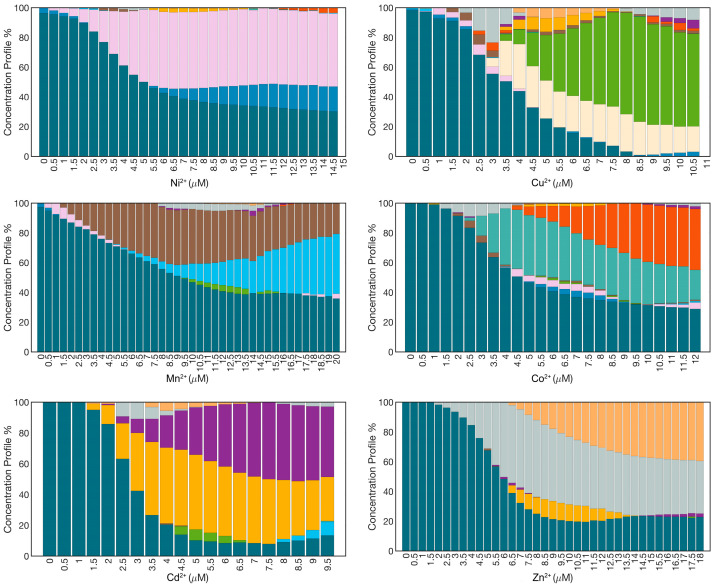
Concentration profiles of the chemical species present in the nanoparticle suspension at increasing concentrations of Ni^2+^, Cu^2+^, Mn^2+^, Co^2+^, Cd^2+^, and Zn^2+^. In each panel, individual species are represented by a different color (AgNPs@11MUA: petroleum green; Ni^2+^: blue and pink (primary); Cu^2+^: sand and green (primary); Mn^2+^: light blue and brown (primary); Co^2+^: pale cyan (primary) and orange; Cd^2+^: yellow (primary) and purple; Zn^2+^: gray (primary) and light orange).

As evident from Figure 1, in the absence of further analyses, the metal ion discriminating power of the AgNPs@11MUA-based sensor is significant only at the end of the titration (before the collapse). Indeed, the extensive superimposition of other regions of the spectra makes it challenging to extract accurate information about the absorption profile of each metal ion. To address this issue, we employed multivariate curve resolution–alternating least squares (MCR-ALS) analysis. MCR-ALS is a powerful chemometric technique commonly used to resolve complex mixture spectra into their pure components and corresponding concentration profiles. The submission of the spectrophotometric raw data to MCR-ALS tool helped in dissecting the concentration profiles of pure spectra at each titration step (Figure 2). Specifically, in the absence of any titrating agent, the algorithm identifies the signal derived solely from nanoparticles consisting of 100% of a single pure component (uncoordinated AgNPs, the only species present in all the spectra investigated, which decrease with the increasing concentration of each metal ion). As the concentration of a given ion increases, we can observe the formation of two additional pure components, corresponding to two major cluster types of AgNPs@11MUA-M^2+^, that differ in the number of bridging ions and, consequently, in size/shape [18].

**Figure 3:**
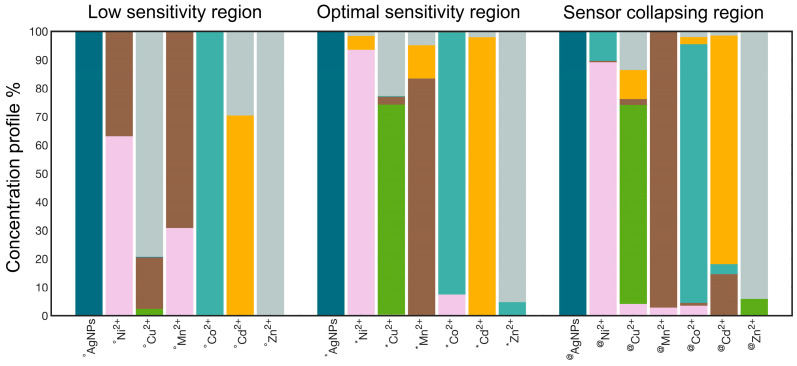
MCR-ALS testing of UV-Vis spectra for each metal ion at (°) the beginning of the aggregation, (*) the central aggregation zone, and (@) the end of the aggregation process, respectively. Individual species are represented by a different color (AgNPs@11MUA: petroleum green; Ni^2+^: pink; Cu^2+^: green; Mn^2+^: brown; Co^2+^: pale cyan; Cd^2+^: yellow; Zn^2+^: gray).

Next, the MCR-ALS model’s ability to recognize unknown SPABs was evaluated by submitting three UV-Vis spectra for each metal ion, with (°) corresponding to the beginning of the aggregation (low-sensitivity region), (*) the central aggregation zone (optimal sensitivity region) and (@) the end of the aggregation process (sensor collapsing region), respectively (Figure 3) [18].

**Figure 4:**
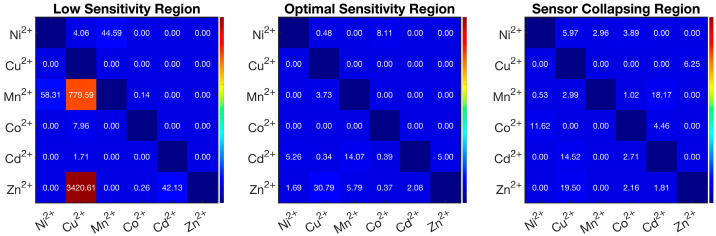
Comparative heatmap visualization of the percentage error of the MCR-ALS model applied to the AgNPs@11MUA sensor under three different ranges of concentrations of individual metal ions. Analyzed ions are listed on the x axis; misidentified ions are listed on the y axis. Percentage scores higher than 10% were considered significant.

Figure 4 shows the heatmap representation of the percentage ratio between incorrect and correct predictions (*R%*) of a target metal calculated as

(3) $$R\%=\left(\frac{Incorrect\;prediction\;of\;the\;target\;metal}{Correct\;prediction\;of\;the\;target\;metal}\right)\times 100$$

The global analyses of the predicted concentration profiles (Figure 3) and of the heatmap (Figure 4) obtained in the low-sensitivity region show an extremely high percentage error in the identification of Cu^2^⁺, which is misidentified as Mn^2^⁺ (R% > 779) and Zn^2^⁺ (R% > 3400), as well as a moderate percentage error (R% > 42) for Ni^2^⁺, Mn^2+^ and Cd^2^⁺, which are misidentified as Mn^2+^, Ni^2+^, and Cd^2+^, respectively.

In the optimal sensitivity region, the error matrix indicated the best predictive ability of the model with the only exception of Cu^2^⁺, which was misidentified as Zn^2^⁺, and Mn^2+^, which was misidentified as Cd^2+^ (14 < R% < 31). These results emphasized the model’s ability to accurately identify individual metal types based on their spectra under optimal conditions. Comparatively, the heat map obtained for the sensor collapsing region evidenced percentage errors never exceeding 20% (the misidentification of Ni^2+^ with Co^2+^, Cu^2+^ with Zn^2+^ and Cd^2+^, and Cd^2+^ and Mn^2+^).

Finally, MCR-ALS was used for the identification of metal ions in complex mixtures from their cumulative absorbance spectra. This method, which was previously trained on the individual absorbance spectra of the metal ions of interest, successfully resolved spectral contributions for most components in complex mixtures (Table 1).

Nevertheless, the cumulative identification of metal ions in mixtures showed reduced sensitivity compared with the identification of individual components from pure solutions. In fact, only at higher concentrations of each metal ion (2.1 μM for Ni^2+^, 1.6 μM for Cu^2+^, 3 μM for Mn^2+^, 2 μM for Co^2+^, 1.6 μM for Cd^2+^, and 2.7 μM for Zn^2+^), MCR-ALS successfully resolved overlapping spectra and correctly identified the metal ions present. However, for mixtures containing lower concentrations of metal ions, some components were not detected. For example, Co^2^⁺ in concentrations lower than 2.0 μM was not detected, whereas Cu^2^⁺ and Cd^2^⁺ were not detected at concentrations lower than 0.75 μM. This limitation was attributed to the masking effects of overlapping spectra, to the reduced contribution of weaker absorbers to the overall signal and to the possible formation of hybrid AgNP aggregates with more than a single type of bridging ion.

## 4. Conclusions

Functionalized silver nanoparticles are well-established colorimetric sensors for the detection of metal ions in aqueous solutions. In a previous study, we demonstrated that by tuning the functionalization density of the AgNP surface, we could expand the applicability range while simultaneously decreasing the sensor’s sensitivity.

In this work, we combined the partially functionalized AgNPs@11MUA with the MCR-ALS statistical approach to enhance sensitivity and maximize the sensor’s overall discriminating power. Regarding the identification of each metal ion in pure solutions, the predictive performance of the model was evaluated across three distinct concentration ranges of metal ions, corresponding to the differential sensitivity region of the sensor. In the low-sensitivity region, the model showed limited specificity and sensitivity, with a high rate of prediction errors. In contrast, in the optimal sensitivity region, corresponding to the sensor’s optimal operating zone, it exhibited excellent specificity and sensitivity, ensuring accurate metal ion discrimination and quantification at levels as low as 0.5–2 μM. In the sensor collapsing region, the model maintained good specificity but demonstrated reduced sensitivity, limiting its ability to detect small variations in concentration.

When applied to complex mixtures, MCR-ALS successfully identified most metal ions by deconvoluting the cumulative absorbance spectra. The detection of weaker components, especially in the mixtures containing lower concentrations of certain ions, was occasionally inconsistent, with some metals being undetected.

This synergistic approach of experimental methods and advanced statistical analyses improved the sensor’s discriminative capacity and provided a promising tool for environmental monitoring and analytical applications.

Nevertheless, our results highlight the importance of adapting sensor functionality to specific concentration ranges of the metal ions and emphasize both the potential of combining experimental techniques with advanced statistical tools to improve analytical performance and the need for the further optimization of the overall sensing system to increase its sensitivity for the detection of low-concentration components in complex mixtures.

## Figures and Tables

**Table 1:** The ability of the MCR-ALS model applied to the AgNPs@11MUA sensor to identify each metal ion constituent in complex mixtures under three different ranges of concentrations.

Metal Ion	Low Concentration	Intermediate Concentration	High Concentration
Ni^2+^	detected	detected	detected
Cu^2+^	undetected	detected	detected
Mn^2+^	detected	detected	detected
Co^2+^	undetected	undetected	detected
Cd^2+^	undetected	detected	detected
Zn^2+^	detected	detected	detected

## Data Availability

The completed data of this study are available from the corresponding author upon reasonable request.

## Supplementary Materials

The following supporting information can be downloaded at: https://www.mdpi.com/article/10.3390/nano15010057/s1, Figure S1: Titration curves of AgNPs@11MUA with individual metal ions; Figure S2: Calibration curves for individual metal ions of interest. Figure S3. Hill plot for at AgNPs@11MUA binding to increasing concentrations of Co^2+^. Figure S4. Hill plot for at AgNPs@11MUA binding to increasing concentrations of Ni^2+^. Figure S5. Hill plot for at AgNPs@11MUA binding to increasing concentrations of Cd^2+^. Figure S6. Hill plot for at AgNPs@11MUA binding to increasing concentrations of Cu^2+^. Figure S7. Hill plot for at AgNPs@11MUA binding to increasing concentrations of Mn^2+^. Figure S8. Hill plot for at AgNPs@11MUA binding to increasing concentrations of Zn^2+^. Table S1: Linearity range, detection and of quantification limits for the individual metal ions of interest.
