# Supplementary material for: Discriminating Analysis of Metal Ions via Multivariate Curve Resolution–Alternating Least Squares Applied to Silver Nanoparticle Sensor
Source: Nanomaterials (Basel). 2025 Jan 2;15(1):57. doi: 10.3390/nano15010057 (PMC11723296; doi:10.3390/nano15010057)
Supplement: Supplementary file 1 [file nanomaterials-15-00057-s001.zip › nanomaterials-3373949-Supplementary Materials.pdf]

Supplementary materials

Discriminating analysis of metal ions via multivariate curve resolution–alternating least squares applied to silver nanoparticle sensor

Andrea Rossi [1,2], Massimiliano Cuccioloni [3,*], Francesco Pellegrino [1], Rita Giovannetti [2], Eugenio Alladio [1]

[1] Department of Chemistry and NIS Centre, University of Torino, Via Giuria 7, Torino, 10125, Italy; francesco.pellegrino@unito.it, eugenio.alladio@unito.it, an.rossi@unito.it.
[2] School of Science and Technology, Chemistry Division, University of Camerino, 62032 Camerino, Italy; rita.giovannetti@unicam.it, andrea.rossi@unicam.it.
[3] School of Biosciences and Veterinary Medicine, University of Camerino, 62032 Camerino, Italy; massimiliano.cuccioloni@unicam.it.
* Corresponding Author

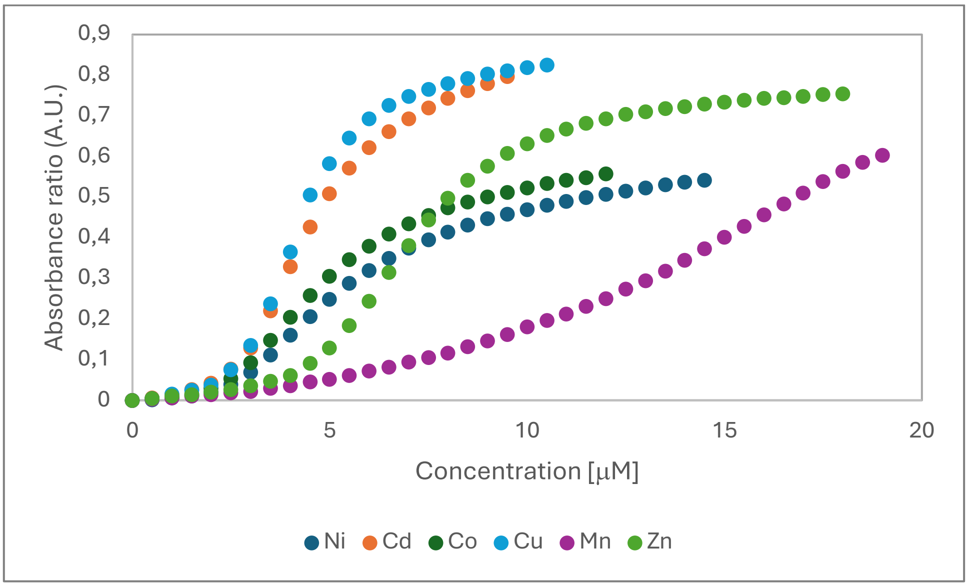

Figure S1. Comparison of absorbance ratios upon titration of partially functionalized AgNPs@11MUA with individual metal ions.

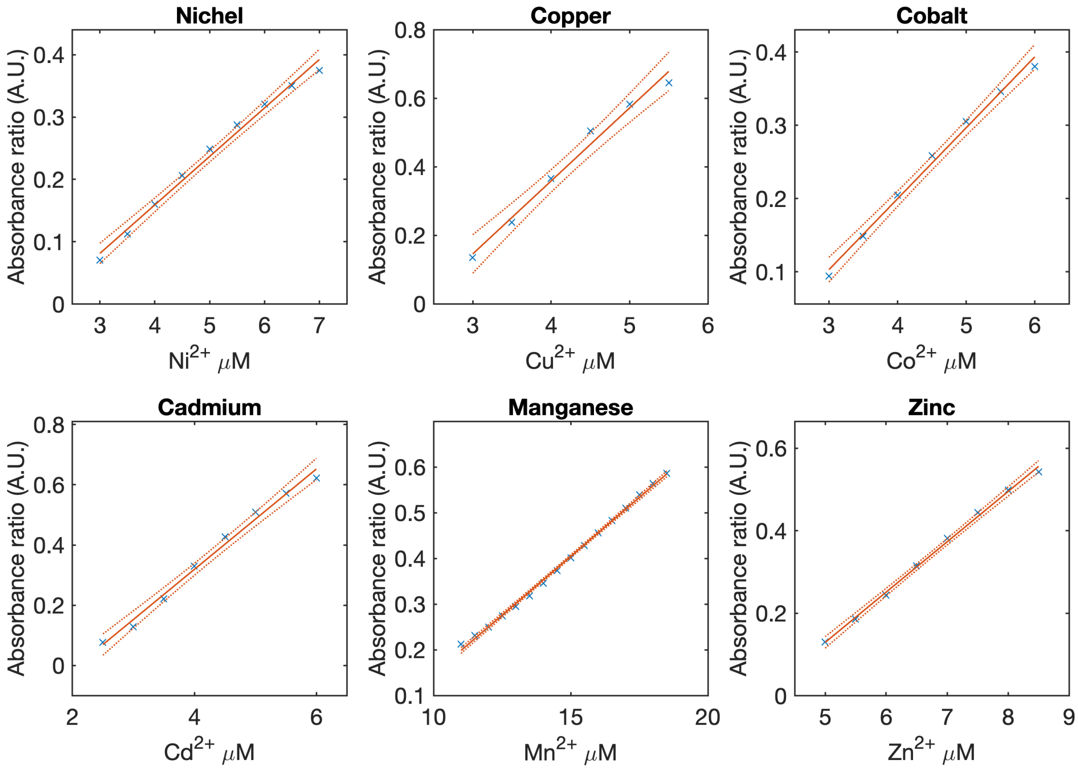

Figure S2. Calibration curves for individual metal ions of interest. Linear fit (red solid lines) and 95% confidence bound (dashed lines) are reported.

Table S1. Linearity range, detection and of quantification limits for the individual metal ions of interest according to the proposed AgNPs@11MUA sensor.

Metal ion	Linearity range (μM)	R^2	LOD (μM)	LOQ (μM)
Ni^{2+}	3-7	0.9903	0.683	2.275
Cu^{2+}	3-6	0.9844	1.136	3.786
Co^{2+}	3-6	0.993	0.669	2.231
Cd^{2+}	2-6	0.9897	0.671	2.237
Mn^{2+}	11-18	0.9976	0.632	2.107
Zn^{2+}	5-8.5	0.997	0.561	1.869

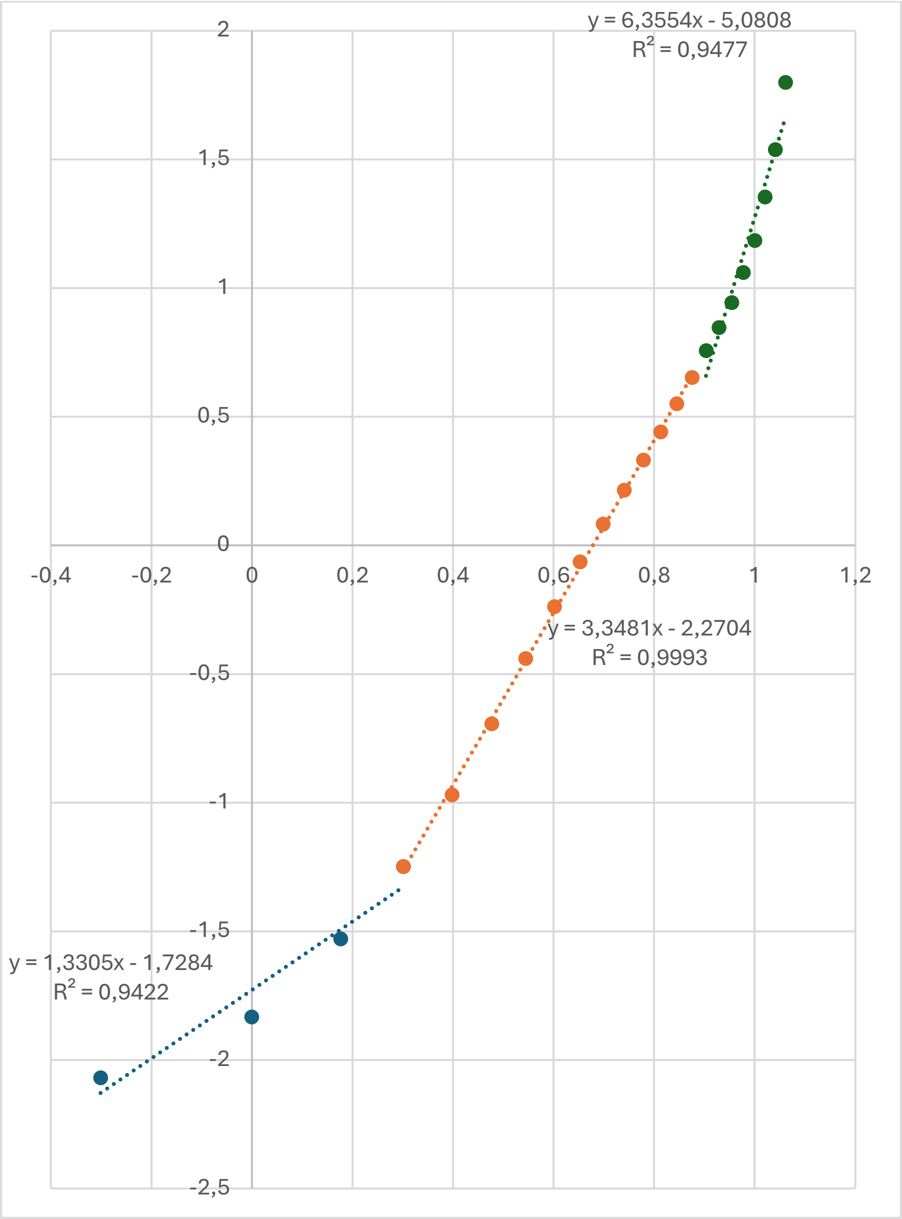

Figure S3. Hill plot for at AgNPs@11MUA binding to increasing concentrations of Co^{2+}.

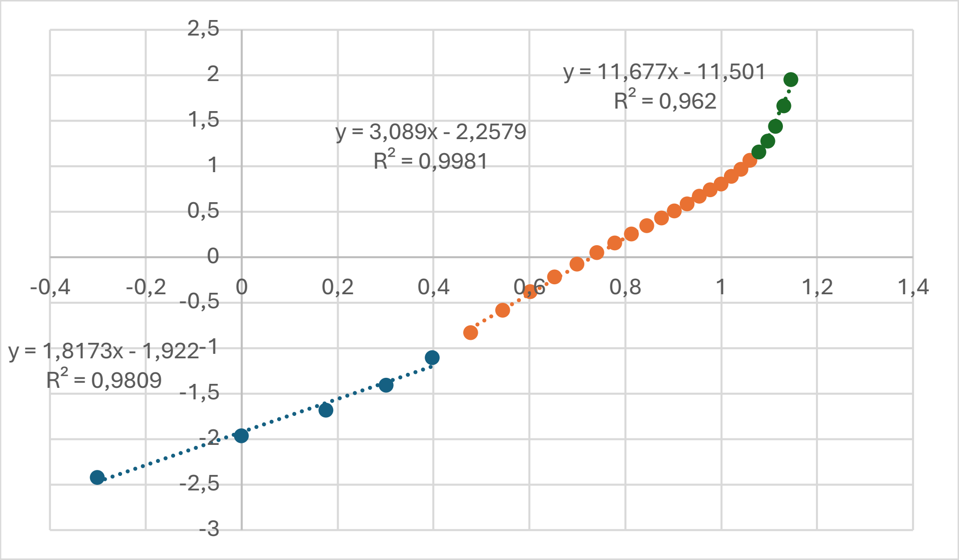

Figure S4. Hill plot for at AgNPs@11MUA binding to increasing concentrations of Ni^{2+}.

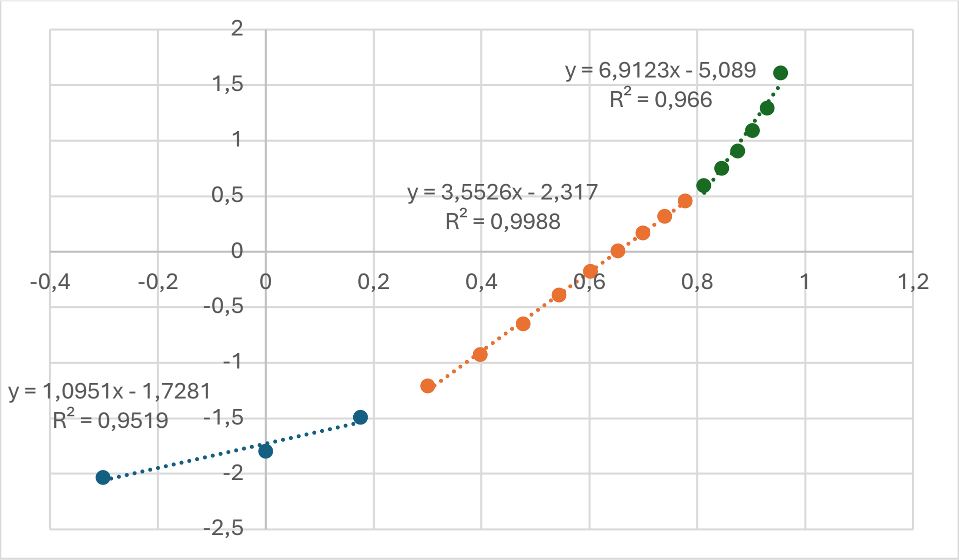

Figure S5. Hill plot for at AgNPs@11MUA binding to increasing concentrations of Cd^{2+}.

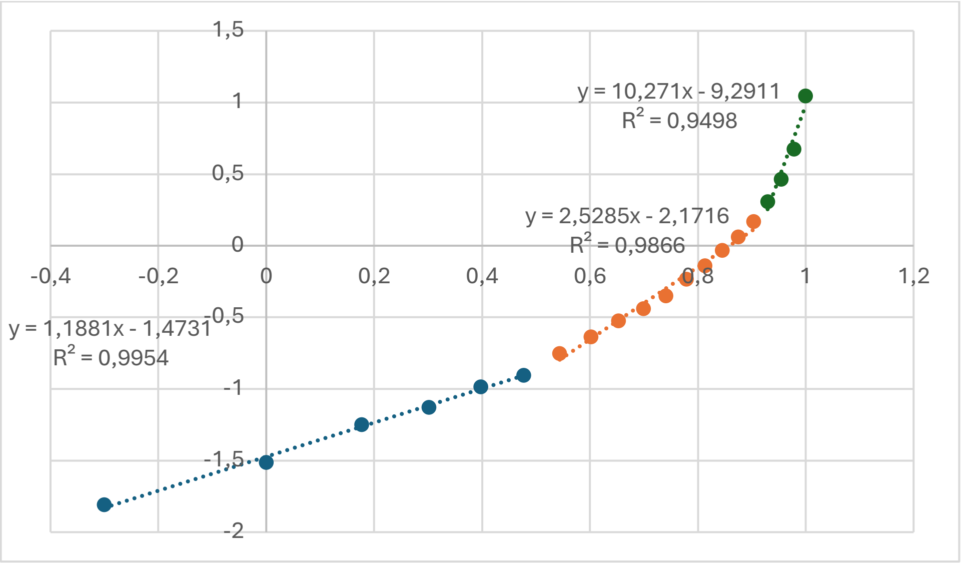

Figure S6. Hill plot for at AgNPs@11MUA binding to increasing concentrations of Cu^{2+}.

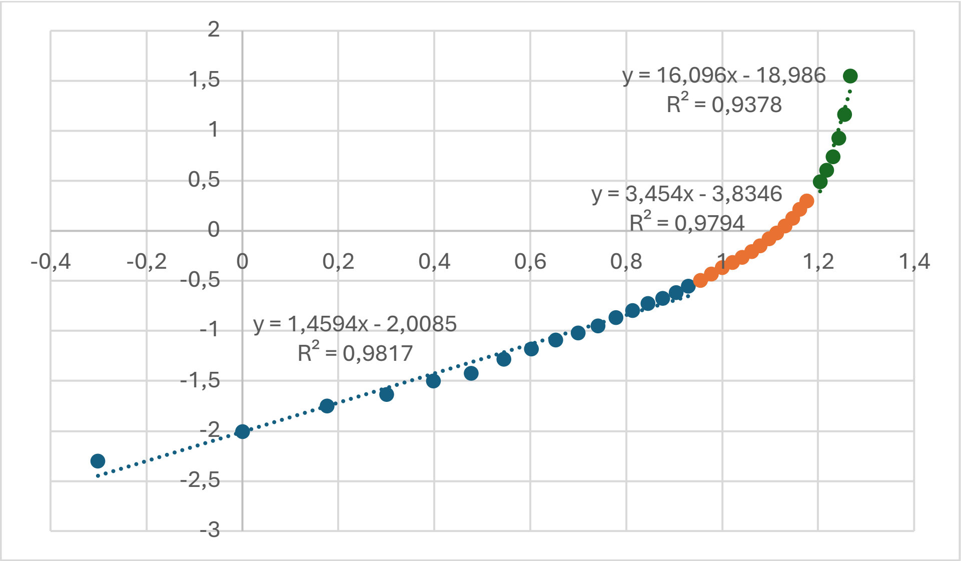

Figure S7. Hill plot for at AgNPs@11MUA binding to increasing concentrations of Mn^{2+}.

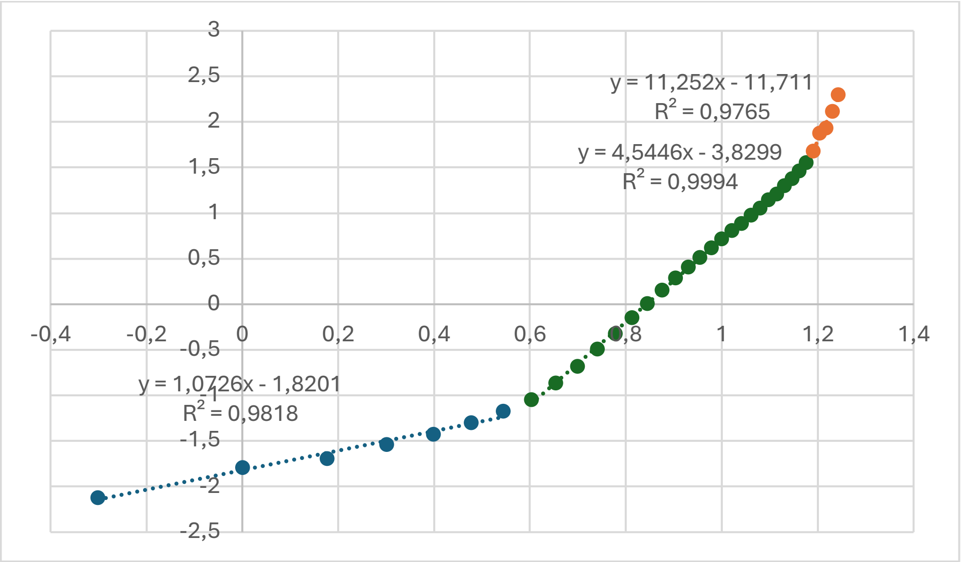

Figure S8. Hill plot for at AgNPs@11MUA binding to increasing concentrations of Zn^{2+}.
